# Recent Trends on Biosurfactants With Antimicrobial Activity Produced by Bacteria Associated With Human Health: Different Perspectives on Their Properties, Challenges, and Potential Applications

**DOI:** 10.3389/fmicb.2021.655150

**Published:** 2021-04-23

**Authors:** Alessandra De Giani, Jessica Zampolli, Patrizia Di Gennaro

**Affiliations:** Department of Biotechnology and Biosciences, University of Milano-Bicocca, Milan, Italy

**Keywords:** human microbiota, human health, bioactive molecules, biosurfactants, antimicrobial activity, -*omic* approach

## Abstract

The attention towards the bacteria associated with human health is growing more and more, above all regarding the bacteria that inhabit the niches offered by the human body, i.e., the gastrointestinal tract, skin, vaginal environment, and lungs. Among the secondary metabolites released by microorganisms associated with human health, little consideration is given to the biosurfactants, molecules with both hydrophobic and hydrophilic nature. Their role in the complex human environment is not only the mere biosurfactant function, but they could also control the microbiota through the *quorum sensing* system and the antimicrobial activity. These functions protect them and, accordingly, the human body principally from microbial and fungal pathogens. Consequently, nowadays, biosurfactants are emerging as promising bioactive molecules due to their very different structures, biological functions, low toxicity, higher biodegradability, and versatility. Therefore, this review provides a comprehensive perspective of biosurfactants with antimicrobial activity produced by bacteria associated with the human body and related to everything human beings are in contact with, e.g., food, beverages, and food-waste dumping sites. For the first time, the role of an “*-omic*” approach is highlighted to predict gene products for biosurfactant production, and an overview of the available gene sequences is reported. Besides, antimicrobial biosurfactants’ features, challenges, and potential applications in the biomedical, food, and nutraceutical industries are discussed.

## Introduction

Innumerable symbiotic, pathogenic, and commensal microbes colonized the human body collectively acknowledged as human microbiota (Malla et al., 2019).

**FIGURE 1:**
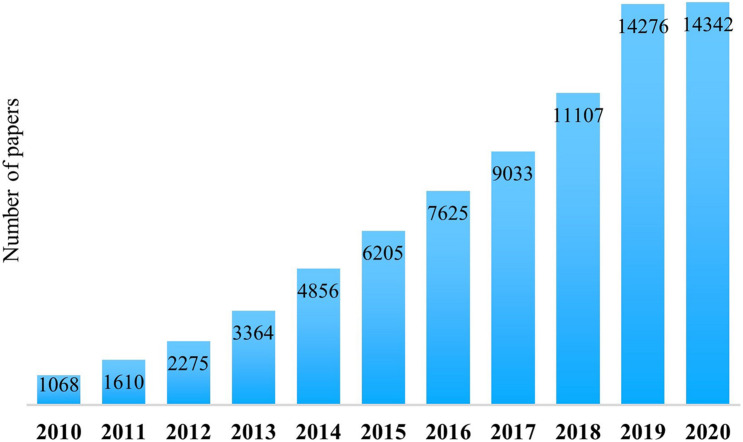
Number of published papers in PubMed with “microbiota” as a keyword, considering a 10-year period (from 2010 to 2020).

Nowadays, interest in the human microbiota, the relative metabolites, and its effects on the host is rapidly growing. In 2010, searching “*microbiota*” within the PubMed database (the free search system accessing the MEDLINE database of references and abstracts about life science and biomedical studies) accounted for only 1,068 items. In 2020, 14,342 items were published and collected under the same keyword (Figure 1). This significant explosion is because of the recent awareness about the essential functions carried out by human-associated microorganisms and the importance of their secondary metabolites for host physiology. Indeed, both bacteria and their metabolites have a role in host metabolism, immune system’s reactivity, neuronal development, and well-being (Zmora et al., 2019).

The human microbiota is composed of 10–100 trillion bacteria living in symbiosis with us (Ursell et al., 2012), mostly belonging to four phyla: Actinobacteria (36.6%), Firmicutes (34.3%), Proteobacteria (11.9%), and Bacteroidetes (9.5%) (Reid et al., 2011). Microbial communities inhabit any accessible area of a host body; nevertheless, the niches with stable communities in humans include the respiratory system, nasal and oral cavities, skin, vagina, and urinary tract, and gastrointestinal (GI) system (Barton et al., 2019). Among the body sites, the intestine receives more attention because it harbors several interesting bacteria, like lactic acid bacteria (LAB). For instance, members of the *Lactobacillus* genus are the most prominent LAB to promote human health due to their ability to produce several bioactive molecules, such as hydrogen peroxide (H_2_O_2_), short-chain fatty acids (SCFAs), and bacteriocins (Satpute et al., 2016).

In general, human-associated bacteria can also produce surface-active compounds that are useful molecules for biomedical and biotechnological applications. Biosurfactants (BSs) are surface-active compounds characterized by a double nature, both hydrophilic (polar) and hydrophobic (non-polar) (Ghasemi et al., 2019). These molecules can have a role in the maintenance of microbial homeostasis, primarily in the oral cavity and vagina (Reid et al., 2011). BSs can be industrially employed in different fields because of their properties—high biodegradability, environmentally and eco-friendly—several have low toxicity against human hosts and resistance at extreme conditions of pH and temperature maintaining their activity (Santos et al., 2016).

Besides, surface-active compounds can exhibit different activities such as antiadhesive, antiviral, anticancer, anti-HIV, anti-inflammatory immune-modulatory, and antimicrobial activities (Banat et al., 2000; Seydlová and Svobodová, 2008; Jahan et al., 2020).

Additionally, they can be produced from the bacterial fermentation of natural waste products, promoting low-cost bioprocesses (Ghasemi et al., 2019).

A large number of reviews have detailed the promising applications of BS molecules from different bacteria to resolve biomedical problems or for food preservation (Fariq and Saeed, 2016; Naughton et al., 2019; Satpute et al., 2019; Ribeiro et al., 2020). Few review papers inquire into the human body sites where BSs are produced as well as the diverse bioactivities that they harbor (beyond the surfactant capacity) (Chen et al., 2017). Among the other activities, no articles report a collection of surface-active compounds with an antimicrobial activity deriving from bacteria associated with human health. Furthermore, no reviews associated antimicrobial BSs with an “-*omic*” approach to unveil the biosynthetic genes that may encode pathways capable of producing specialized metabolites.

This review focuses on the antimicrobial BSs principally produced by bacteria associated with human health; specifically, we refer to the human microbiota and food-associated bacteria (including bacteria derived from food and beverage or environmental sites related to food such as rhizosphere of agricultural fields or kitchen waste dumping sites). In detail, we will discuss antimicrobial BS structures and chemical properties in relation to the exerted activities, especially the antimicrobial activity. Besides, this work represents the first attempt to correlate the antimicrobial BSs with the genes harbored by the bacteria possibly involved in the production of antimicrobial BSs. Moreover, BS features will be correlated to the possible beneficial effects for the host, and the potential industrial and biotechnological applications.

## Biosurfactant Produced by Bacteria Associated With Human Health

The stability and the correct balance of the microbial communities of the different human body sites are strictly correlated with the surface-active compounds that they produce. Ding and Schloss (2014) reported that there are correlations between communities placed in different body sites, like the oral cavity and the vagina, but also between stool samples and the oral cavity. Therefore, they affirmed that bacteria could pass through the gut and the intestine and share some ecological environments. The most unstable microbiota belongs to the oral cavity, while the most stable are placed in stool and vagina. Accordingly, the release of bioactive molecules including BSs could be crucial for the maintenance of a niche. For example, the health status of the vaginal environment is associated with a stable community of lactobacilli. However, *Lactobacillus* strains with a BS-producing activity are not prevalent in the vaginal environment, and BS molecules could disseminate in the milieu and change the surface tension (ST) to block the pathogens (Banat et al., 2010).

Members of the *Lactobacillus* genus can produce BSs composed principally of protein, polysaccharide, and phosphate in different ratios (Brzozowski et al., 2011), and they are mainly classified as glycolipids or glycolipoproteins (Fracchia et al., 2010; Morais et al., 2017; Supplementary Table S1). The molecules have also an antimicrobial effect against several common potential pathogenic bacteria such as *Neisseria gonorrhoeae* (Foschi et al., 2017), *Escherichia coli*, *Staphylococcus saprophyticus*, *Enterobacter aerogenes*, and *Klebsiella pneumoniae* and an antifungal activity against *Candida albicans* (Morais et al., 2017). Nevertheless, lactobacilli are present in other compartments of the human body, i.e., the skin, oral cavity, intestine, and gut.

The GI tract usually possesses a stable microbiota community, but several BS-producing bacteria were isolated from food, and only their intake allows them to inhabit or transit in it. For instance, *Lactobacillus paracasei* ssp. *paracasei* A20 was isolated from Portuguese dairy plants; and the produced cell-free BS has potent antimicrobial and antiadhesive activities against several bacteria and fungi (Gudina et al., 2010). Also, *Lactobacillus acidophilus*, *Lactobacillus pentosus*, and *Lactobacillus fermentum* isolated from dairy products, breast milk, fermented shrimps, and fruits in Malaysia produce cell-free BSs with an antimicrobial activity (Abdalsadiq and Zaiton, 2018). Among the other BS producers, *Pediococcus dextrinicus* SHU1593 (re-classified as *Lactobacillus* in Haakensen et al., 2009) produces a cell-bound lipoprotein BS with an antimicrobial activity against *Bacillus cereus*, *E. aerogenes*, and *Salmonella typhimurium* (Ghasemi et al., 2019; Supplementary Table 1).

Concerning the oral cavity, Merghni et al. (2017) suggested the use of the cell-associated BS molecules produced by *Lactobacillus casei* LBI and *L. casei* American Type Culture Collection (ATCC) 393 for the prevention of oral diseases since their antimicrobial and antibiofilm activities against *Staphylococcus aureus* (Supplementary Table 1).

The ability to produce antimicrobial BSs is evidenced not only for bacteria belonging to *Lactobacillus* genus but also for other inhabitants of different sites of the human body. Among them, *Pseudomonas aeruginosa* is a notable producer of BSs; and for example, *P*. *aeruginosa* ATCC 10145 is able to produce a cell-free rhamnolipid BS with antimicrobial and antifungal activities (El-Sheshtawy and Doheim, 2014; Supplementary Table 1).

In the literature, the major class of antimicrobial BSs from bacteria associated with human health is lipopeptides, glycolipids, glycopeptides, and glycolipoproteins (Abdalsadiq et al., 2018; Abdalsadiq and Zaiton, 2018; Hippolyte et al., 2018; Emmanuel et al., 2019). About 16 research papers described lactobacilli antimicrobial BS production, 10 of which are characterized as cell-free BSs and seven as cell-associated BSs (Cornea et al., 2016) describe two kinds of molecules, one released in the medium and one bound to the cell. Among the cell-free BSs, five described only the nature of the released molecules including small glycolipids as in the case of the molecule released by *L*. *acidophilus* NCIM 2903 (Satpute et al., 2018) and lipopeptides, i.e., the one produced by a *Lactobacillus* strain isolated from homemade curd (Emmanuel et al., 2019). Instead, the cell-associated antimicrobial BSs are more complex and characterized by several constituents. For example, many papers described glycolipoproteins (Morais et al., 2017; Hippolyte et al., 2018; Satpute et al., 2019; Supplementary Table 1). However, details about conformations, or molecular weight, or mechanisms of action are still unknown.

## Prediction of Biosurfactant With an Antimicrobial Activity by an “*-omic*” Approach

Several research studies are underway to find new BSs and antimicrobial compounds that can be used for biotechnological and medical applications or to fight against resistant pathogens. However, the efforts often lack the understanding of the molecular mechanisms that are behind their production, since the primary approaches usually involve several cultivation conditions and experimental assays before understanding which kind of secondary metabolites are produced by a microorganism (Gomaa, 2013; Hippolyte et al., 2018; Emmanuel et al., 2019).

The advent of the so-called “-*omic era*,” in other words, in the last two decades when research studies were mainly undertaken on a genome-wide scale, changed the perspective (Zampolli et al., 2018). Together with the development of new bioinformatics tools, the amount of available bacterial genome sequences that allow the reconstruction of biosynthetic gene clusters (BGCs) that may encode pathways capable of producing specialized metabolites has been growing (Ceniceros et al., 2017). Therefore, the search for new antimicrobial compounds and BS molecules could begin with the exploration of a strain genome.

**FIGURE 2:**
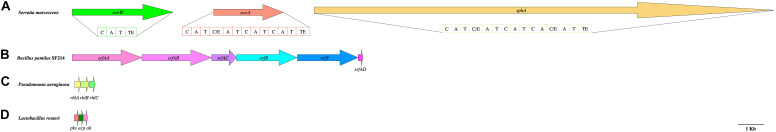
Gene clusters involved in the biosynthesis of biosurfactant with antimicrobial activity. **(A)** Serrawettin genes from *Serratia marcescens*—*swrW*, *swrA*, and *sphA*—encoding, respectively, for serrawettin W1, serrawettin W2, and stephensiolide. The gene domains are as follows: C, condensation; A, adenylation; T, thiolation; TE, thioesterase. **(B)** *srf* operon [non-ribosomal peptide synthetase (NRPS) family] from *Bacillus pumilus* SF214—*srfAA*, *srfAB*, and *srfAC*—encoding, respectively, for surfactin synthase subunit 1, 2, and *orfX*; *orfY* encoding for uncharacterized genes; and *srfAD* encoding for surfactin synthase thioesterase. **(C)** Rhamnolipid genes from *Pseudomonas aeruginosa* CR1—*rhlA*, *rhlB*, and *rhlC*—encoding, respectively, for chain A of a rhamnosyl transferase, chain B of the enzyme, and rhamnosyl transferase 2. **(D)** Type II polyketide synthase genes (PKS) from *Lactobacillus reuteri*—*pks*, *acp*, and *ah* genes—encoding for PKS condensation, β-ketoacyl-[ACP] synthase II, and Ab hydrolase, respectively.

Referring only to the number of papers describing antimicrobial BSs produced by bacteria associated with human health (including human microbiota and bacteria from food, food-waste dumping sites, and agricultural soil for food production), few so far have exploited an “-*omic*” approach and studied the genetic determinants (Table 1). Considering only these articles, until now, the genera associated with biosynthetic genes and BGCs are the following: *Serratia*, *Bacillus*, *Pseudomonas*, and *Lactobacillus* (Figure 2).

**TABLE 1:** List of available genes involved in the biosynthesis of biosurfactant with antimicrobial activity produced by bacteria associated with human health.

**Gene**	**Product**	**Strain**	**References**
*swrW*, *swrA*, *sphA* (NRPS family)	Non-ribosomal serrawettin W1 synthetase (*swrW*), serrawettin W2 synthetase (*swrA*), stephensiolides (*sphA*)	*Serratia marcescens*	Clements et al., 2019a, b
*sfp*	Putative surfactin transcriptional terminator (*sfp*)	*Bacillus amyloliquefaciens*, *Bacillus thuringiensis*, *Bacillus subtilis*	Perez et al., 2017; Isa et al., 2020
*srfAA*, *srfAB*, *srfAC*, and *srfAD* (NRPS family)	Surfactin synthase subunit 1, 2, and 3 (*srfAA*, *srfAB*, *srfAC* surfactin synthase thioesterase subunit *srfAD*)	*Bacillus pumilus* SF214	Saggese et al., 2018
*rhlA*, *rhlB*, and *rhlC*	Chain A of a rhamnosyl transferase (*rhlA*), chain B of the enzyme (*rhlB*), rhamnosyl transferase 2 (*rhlC*)	*Pseudomonas aeruginosa* CR1	Sood et al., 2020
Type II polyketide synthase genes (PKS)	Ketasynthase domain: PKS condensation, β-ketoacyl-[ACP] synthase II, Ab hydrolase	*Lactobacillus reuteri*	Vazquez, 2013
*npsA* (NRPS family)	Unknown molecule with 5289 amino acid length	*Lactobacillus plantarum* WCFS1 and *L. plantarum* RI-515	Vazquez, 2013

*NRPS, non-ribosomal peptide synthetase; PKS, polyketide synthase.*

The most common species of *Serratia* genus is *Serratia marcescens*, whose members are often opportunistic pathogens associated with nosocomial infections, such as the urinary and respiratory tracts, surgical wound, and bloodstream infections (Khanna et al., 2013). On the contrary, environmental *Serratia* strains including food-associated bacteria are non-pathogenic strains (Sandner-Miranda et al., 2018); for this reason, this category is included in the considered antimicrobial BS producers related to humans.

Biosurfactants produced by members of the *Serratia* genus are low-molecular-weight, non-ionic lipopeptides with an antimicrobial activity comprising serrawettin W1, W2, W3, and stephensiolides A to K (cyclic lipopeptides characterized by a macrolactone ring) (Clements et al., 2019a). The investigation into the mechanisms involved in the biosynthesis of serrawettin W1, serrawettin W2, and stephensiolides revealed *swrW*, *swrA*, and *sphA* genes, respectively (Figure 2A). In the open reading frame (ORF), modules consist of condensation, adenylation, thiolation, and thioesterase domains (Clements et al., 2019a; Figure 2A). These discoveries have been already exploited by Clements et al. (2019b) for the screening of 22 BS-producing bacteria isolated from municipal wastewater treatment plants for the biosynthesis of serrawettin A. They used a primer set designed for the identification of *swrA* gene and *swrW* gene, encoding for the non-ribosomal serrawettin W2 synthetase and serrawettin W1 synthetase, respectively.

The same approach has also been applied to discover new antimicrobial BSs in members of the *Bacillus* genus. For instance, *Bacillus subtilis* produces a lipopeptide BS known as surfactin, which is characterized as a cyclic heptapeptide linked to a β-hydroxy fatty acid (Perez et al., 2017). The surfactin transcriptional terminator is encoded by *sfp* gene, which can be employed as a marker sequence (Perez et al., 2017; Isa et al., 2020). Besides, the *srfA* operon can be used for the prediction of surfactin production by members of the *Bacillus* genus (Kanmani et al., 2013). For example, Saggese et al. (2018) searched for *srfAA*, *srfAB*, *srfAC*, and *srfAD* genes, encoding respectively for surfactin synthase subunit 1, 2, 3, and surfactin synthase thioesterase (Figure 2B).

Interestingly, both *srw* genes of *Serratia* and *srfA* genes of *Bacillus* are considered part of the non-ribosomal peptide synthetase (NRPS) family (Saggese et al., 2018; Clements et al., 2019a), comprising multi-modular enzyme complexes essential for the synthesis of secondary metabolites, such as antibiotics (Singh et al., 2017).

Another gene cluster useful as a marker sequence for the search of BSs with an antimicrobial activity is the *rhl* operon discovered in members of the *Pseudomonas* genus, encoding for rhamnolipid production. The gene cluster comprises the following: *rhlA* gene encoding for chain A of a rhamnosyl transferase, utilizing ACP-βhydroxy-acids and producing a fatty acid dimer; *rhlB* gene encoding for chain B of the same enzyme, using the fatty acid dimer and TDP-L-rhamnose as the substrate to catalyze the formation of mono-rhamnolipids; and *rhlC* gene encoding for a rhamnosyl transferase 2 that produces di-rhamnolipids from mono-rhamnolipids and a rhamnose moiety (Sood et al., 2020; Figure 2C). It is interesting to note that *rhlAB* operon is under the control of factors related to the *quorum sensing* system. Consequently, it undergoes transcriptional and post-transcriptional regulations; and the transcription depends on the environment (Reis et al., 2011). It is noteworthy to underline that Sood et al. (2020) used *in silico* analysis to predict rhamnolipid biosynthetic pathways before the extraction of the BS from *Pseudomonas* sp. CR1.

Type II polyketide synthase genes (PKS) is a well-characterized gene family involved in the production of antimicrobial BS molecules. The gene products are enzymes for the biosynthesis of polyketides composed of various domains; and they are generally grouped with the NRPSs because of the complex biosynthetic machinery and the production of secondary metabolites with an antimicrobial activity (Singh et al., 2017). Most of the KS domains in PKS genes are found in Actinobacteria synthetizing BSs and antimicrobial molecules (Selvin et al., 2016). Nevertheless, the work of Vazquez (2013) described a *Lactobacillus reuteri* strain harboring a single PKS gene cluster including the following gene products: PKS condensation (*pks*), β-ketoacyl-[ACP] synthase II (ACP is acyl carrier protein) (*acp*), and Ab hydrolase (*ah*) (Figure 2D).

Despite the several massive sequencing projects regarding bacteria of *Lactobacillus* genus or intestinal related genera, little is known about the genes encoding for the BS production potential. Vazquez (2013) screened *in silico* the surlactin BS genes from 173 *Lactobacillus* species, indicating with the termed “surlactin” every kind of BS produced by lactobacilli, regardless of the chemical nature or if the molecules are cell-bound or released in the medium. In general, they conclude that several strains of *Lactobacillus plantarum*, *Lactobacillus inners*, *L*. *reuteri*, and *Lactobacillus brevis* have the potential to produce surlactin sharing high identity with surfactin biosynthetic pathway (principally from *Bacillus* species; this could be expected because *Bacillus* and *Lactobacillus* genera belong to the Bacillaceae family). Indeed, they found that lactobacilli harbor both NRPS and PKS genes. Specifically, *L*. *plantarum* WCFS1 and RI-515 showed NRPS genes that comprise *npsA* gene encoding for an unknown 5,289-amino acid length chain that is a good candidate as a marker sequence for surlactin production, while the abovementioned *L. reuteri* harbors the PKS genes. Unfortunately, NRPS and PKS systems are connected not only with BS synthesis but also with other secondary metabolites such as antibiotics and bacteriocins (Lin et al., 2015; Zhang et al., 2019). Such overlap of possible transcriptional functions can lead to the prediction of the ability to produce different secondary metabolites.

## Classification of Biosurfactant Compounds From Bacteria Associated With Human Health

The antimicrobial BSs derived from microorganisms associated with human health can be categorized into two main classes: cell-associated and cell-released BSs (Supplementary Table 1).

**FIGURE 3:**
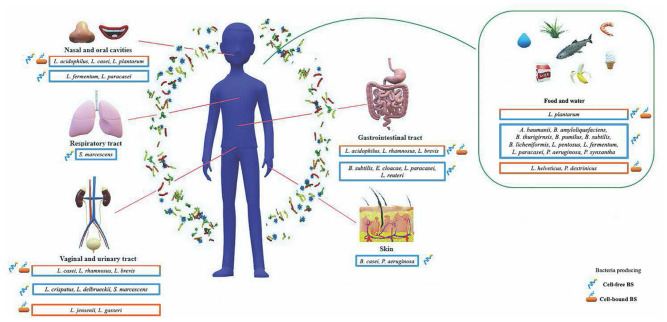
Schematic representation of biosurfactants with antimicrobial activity produced by microorganisms associated with human health according to the human body districts. Bacteria enclosed in an orange box produce biosurfactants (BSs) with antimicrobial activity belonging to the cell-bound class; bacteria enclosed in a light blue box produce BSs with antimicrobial activity released in the surrounding environment; bacteria enclosed in an orange-light blue box produce BSs with antimicrobial activity belonging to both classes.

Literature survey illustrates that the bacteria producing antimicrobial BSs associated with human health mostly belong to the phyla Firmicutes and Proteobacteria. Currently, the bacteria genera grouped within cell-associated BS class fall in the genera *Lactobacillus* and *Pediococcus*, while the cell-released BS class includes few strains of *Lactobacillus*, *Pseudomonas*, *Bacillus*, and *Enterobacter* genera (Figure 3).

### Cell-Associated Biosurfactants

The antimicrobial BSs from the *Lactobacillus* genus are generally cell-associated, probably for their intrinsic characteristics. The chemical analysis of characterized cell-associated BSs shows that they are high-molecular-weight molecules composed mainly of proteins, fatty acids, and sugars with different percentages.

Satpute et al. (2019) isolated a glycolipoprotein-type BS from *Lactobacillus* acidophilus NCIM 2903 with the ability to reduce the ST of 45 mN m^–1^ (from 71 to 26 mN m^–1^) and a critical micelle concentration (CMC) equal to 23.6 mg ml^–1^. The antibacterial and antiadhesive properties of the glycolipoprotein were detected at a concentration of 25 mg ml^–1^ that was able to inhibit *Escherichia coli* NCIM 2065 and *Proteus vulgaris* NCIM 2027 growth of more than 30% and *Bacillus subtilis* MTCC 2423 growth of 26%. The action was not so potent against *Pseudomonas putida* MTCC 2467 (14% of growth inhibition). The stronger antiadhesive effect was against two Gram-positive bacteria as shown by 81% and 79% of inhibition of *Staphylococcus aureus* NCIM 2079 and *B. subtilis* MTCC 2423, respectively. Moreover, the cell-associated glycolipoprotein showed antiadhesive and antibiofilm potential against *P*. *vulgaris* NCIM 2027 and *S. aureus* NCIM 2079.

Similarly, antimicrobial BSs from *Lactobacillus jensenii* P_6A_ and *Lactobacillus gasseri* P_65_ isolated from vaginal fluids of healthy women after 72 h of fermentation yield of 0.27 and 0.42 g L^–1^, respectively (Morais et al., 2017). The BSs produced by *L*. *jensenii* P_6A_ and *L*. *gasseri* P_65_ reduced the water-surface tension of 28.8 mN m^–1^ (from 72 to 43.2 mN m^–1^) and 29.5 mN m^–1^ (from 72 to 42.5 mN m^–1^) with comparable CMC values of 7.1 and 8.58 mg ml^–1^, respectively. They also shared a similar chemical composition: P_6A_ molecule was composed of 51.49% of carbohydrates, 15.17% of proteins, and 29.45% of lipids, while P_65_ was composed of 38.61% of carbohydrates, 9.81% of proteins, and 49.53% of lipids. Considering the last constituent category, only 14-methypentandecanoic acid, a 16-carbon fatty acid, was present in both BSs. This molecule was the main fatty acid present in *L. jensenii* P_6A_ BS, representing 69% of the lipid fraction, while eicosanoic acid (47.43% of the lipid fraction) characterized *L. gasseri* P_65_ BS. Furthermore, galactose, glucose, and ribose were present in both the molecules in different percentages, even if rhamnose is peculiar of *L. jensenii* P_6A_. The antimicrobial activity against different potential human urogenital tract pathogens showed similar minimum inhibitory concentration (MIC) values for the two isolated BSs: 16 μg ml^–1^ for *E. coli* and 128 μg ml^–1^ for *Klebsiella pneumoniae*, *Enterobacter aerogenes*, and *Staphylococcus saprophyticus*. Interesting is the antifungal activity against the potential vaginal pathogen *Candida albicans* ATCC 18804, which was inhibited at a concentration of 16 μg ml^–1^. Furthermore, the biomolecules exhibited an antibiofilm activity showing the best result against *E. aerogenes* (its biofilm was disrupted for 64%).

Sambanthamoorthy et al. (2014), Cornea et al. (2016), and Merghni et al. (2017) reported the production of cell-associated antimicrobial BSs from different *Lactobacillus* strains, without any structural characterization. *Lactobacillus casei* produces two cell-associated BSs, named BS-B1 and BS-Z9 with an antioxidant activity (Merghni et al., 2017). At a concentration of 5.0 mg ml^–1^, BS-B1 and BS-Z9 BSs showed 74.6 and 77.3% of α-diphenyl-β-picrylhydrazyl (DPPH) radical scavenging activity, respectively. Furthermore, the antiproliferative potential on human epithelial cells HEp-2 after 48 h showed calculated values of IC_50_ that ranged from 109.1 to 129.7 mg ml^–1^. Moreover, the antiproliferative effect of BSs was directly proportional to its concentration; indeed, at the maximum BS-B1 and BS-Z9 concentration of 200 mg ml^–1^, the antiproliferative levels were 67.19% and 66.72%, respectively. The antimicrobial and antiadhesive activities were evaluated only against *S. aureus* strains (the reference strain ATCC 6538 and the oral strains 9P and 29P). BS-LZ9 showed an antibacterial effect against the ATCC 6538 strain, while BS-B1 was effective against the oral pathogens 9P and 29P, showing IC_50_ values of 1.92 and 2.16 mg ml^–1^, respectively. However, the ATCC strain was more susceptible to the displacing (80.87–84.86% of inhibition) rather than 29P strain (48.74–68.84%) at a concentration of 12.5 mg ml^–1^. The antiadhesive capacity was maintained at the lowest concentration (1.56 mg ml^–1^) since both BSs inhibit the adhesion of *S. aureus* ATCC 6538 and 9P of almost 50%. The antibiofilm potential was effective also on pre-formed biofilms.

Cornea et al. (2016) isolated *Lactobacillus plantarum* L26, *L. plantarum* L35, and *Lactobacillus brevis* L61 from Romanian traditional fermented food for the ability to produce BSs. Their antimicrobial and antifungal activities were evaluated against microorganisms having a role in food contamination or spoilage. All the extracted cell-bound BSs could inhibit *E*. *coli* growth, while a limited inhibition effect was observed by the L61 strain BS against *Bacillus cereus*. No effect was evidenced against *S. aureus*, yeast, and fungi. However, the BSs were able to inhibit the mycotoxigenic fungi sporulation, without affecting the mycelial growth that was justified assuming a non-optimal BS concentration.

Finally, Sambanthamoorthy et al. (2014) focused on *L. jensenii* and *Lactobacillus rhamnosus* able to inhibit clinical multidrug-resistant (MDR) strains of *Acinetobacter baumannii* (AB5075 and AB5711), *E. coli* EC433, and *S. aureus* strains [methicillin-resistant *S. aureus* (MRSA), clinical isolate 243, and UAMS-1]. Their crude BS extracts were effective against the MDR pathogens at a concentration of 50 mg ml^–1^. The best inhibition was due to *L. rhamnosus* molecule whose activity suppressed *A. baumannii* growth of 96–97%, *S. aureus* UAMS-1 and MRSA strains between 80 and 93%, and *E. coli* of 72–85%. These BSs also showed antibiofilm potential in a range of 25 to 50 mg ml^–1^. At the maximum concentration, *A. baumannii* and *E. coli* could not produce biofilm, while the ability was unpaired at lower concentrations for *S. aureus*. In order to use these biomolecules for biomedical applications, the cytotoxicity was tested against human A549 lung epithelial cells with different BS concentrations (from 25 to 200 mg ml^–1^) for 24 h. Both biomolecules were a little bit toxic at the maximum concentration, while they were safe from 25 to 100 mg ml^–1^.

Among other bacteria belonging to the Firmicutes phylum, an antimicrobial cell-bound BS was isolated from the lactic acid bacterium *Pediococcus dextrinicus* SHU1593 (Ghasemi et al., 2019). BS yield of 0.7 g L^–1^ was obtained when the strain grew on three substrates including a modified MRS medium (where glucose was replaced by lactose and Tween 80 was not present) and two low-cost materials such as molasses and date syrup. The BS solution at a CMC of 2.7 mg ml^–1^ showed the minimum ST of 39.01 mN m^–1^. The chemical characterization pointed out its lipoprotein composition with around 52% (w/w) and 47% of lipid and proteins content, respectively (the remaining 1% corresponds to sugars, but the authors attributed this portion to the precipitated culture medium). The predominant fatty acids were oleic (60.28%), palmitic (25.08%), stearic (7.43%), and lauric (4.60%) acids. The BS composition was not dependent on the growth media; thus, they all had similar nature, which was comparable with lipopeptides produced by members of *Bacillus* genus. At a concentration of 25 mg ml^–1^, *Pediococcus* BS inhibited *E*. *coli*, *E*. *aerogenes*, and *Pseudomonas aeruginosa* growth. However, the biomolecule was not active against Gram-positive bacteria such as *B*. *cereus* and *S. aureus*. Nevertheless, an important antiadhesive activity was evidenced against *B*. *cereus* (70.5%), *P*. *aeruginosa* (61.84%), and *Salmonella typhimurium* (58.69%).

### Biosurfactants Released by Bacteria in the Surrounding Environment

Most of the BSs with an antimicrobial activity produced by bacteria associated with human health are molecules in the class of BSs released in the surrounding environment belonging to Firmicutes and Proteobacteria phyla (Supplementary Table 1). Understanding their characteristics, mechanisms of action, and their release might help to elucidate the relationships between bacteria–bacteria and bacteria–environment. Consequently, this knowledge brings along the advantage of the high biotechnological potential. To date, the human-associated antimicrobial BSs described in the literature are included in the following chemical types: lipopeptides, glycolipids, glycoproteins, and glycolipoproteins.

#### Lipopeptide Biosurfactants

A proteinaceous nature is widely common among the molecules with an antimicrobial activity. For example, several antibiotics or bacteriocins have a protein nature, as well as the antimicrobial molecules released by the human body or by other cells.

Therefore, the importance of studying the protein domain through a genome-based or molecular approach for the prediction of the antimicrobial potential is clear, as evidenced by NRPS and PKS gene families (see section “Prediction of Biosurfactant With an Antimicrobial Activity by An “*-omic*” Approach”).

Some BSs with a proteinaceous nature have an antimicrobial potential against Gram-negative and Gram-positive bacteria, and several have also antifungal activities principally against *Candida* spp. Numerous authors reported the potential of cell-released BSs as antimicrobial, antifungal, and antibiofilm agents (Gudina et al., 2010; Gomaa, 2013; Perez et al., 2017). Among members of *Lactobacillus* genus, only two research studies report strains able to release antimicrobial lipopeptide BSs (Abdalsadiq et al., 2018; Emmanuel et al., 2019).

The first study reports the bioactive potential of the lipopeptide fraction compared with the glycolipid fraction isolated from the cell cultures of *L*. *acidophilus* and *Lactobacillus pentosus* against several antagonists such as *Proteus mirabilis*, *S*. *aureus*, *Streptococcus pneumoniae*, *K*. *pneumoniae*, and *C*. *albicans* (Abdalsadiq et al., 2018). The inhibition measured by the agar well diffusion assay (AWDA) on the extracted BS resulted in haloes ranging from 14.00 mm (against *K. pneumoniae*) to 44.00 mm (against *S. aureus*). The quantification of the MIC revealed that the lipopeptide fraction had a stronger antimicrobial effect at lower MIC values ranging from 7.81 μg ml^–1^ (against *P. mirabilis*) to 62.5 μg ml^–1^ (against *K. pneumoniae*), whereas glycolipid fraction from 15.6 to 62.5 μg ml^–1^. Furthermore, the antiadhesive activity against all pathogens showed inhibition percentages ranging from 65% (against *P. mirabilis* at a concentration of 250 μg ml^–1^) to 93% (against *K. pneumoniae* at a concentration of 250 μg ml^–1^) depending on the concentration of the lipopeptide fraction, while the antiadhesive activity of the glycolipid fraction produced a smaller percentage of inhibition (from 45 to 72.7%). Finally, the antibiofilm capacity of the molecules was demonstrated at a concentration of 250 μg ml^–1^, whose effectiveness was up to 100%. Specifically, the lipopeptide fraction evidenced the maximum antibiofilm percentage against *K. pneumoniae* and *P. mirabilis* and the lowest against *S. aureus* (85%).

Another example of antimicrobial lipopeptide BS produced by lactobacilli is the one from a *Lactobacillus* sp. strain isolated from homemade curd, yielding 3.21 g L^–1^ (Emmanuel et al., 2019). It was characterized by the presence of alkene, alkyne groups, and conjugated diene and an emulsification index (E_24_) of 58.1%. The antimicrobial and antibiofilm activities of the BS were tested only against those of *E. coli*. The first showed a comparable antimicrobial activity with respect to sodium dodecyl sulfate (SDS), and the BS inhibited the biofilm of *E. coli*. Indeed, after 6 h, the number of *E. coli* cells forming the biofilm was lower as the lipopeptide concentration increased.

Undoubtedly, one of the most well-known lipopeptide BS producers are members of the *Bacillus* genus (Zhao et al., 2017). Several strains are employed as probiotics because of the formation of spores that survive in extreme conditions, such as low gastric pH. Once in the intestine, spores can germinate; thus, *Bacillus* strains grow and re-sporulate, exerting an antimicrobial activity and other beneficial effects. However, nowadays, the use of *Bacillus* species as probiotics is disputed due to the capability of transferring genes for the antimicrobial resistance to the microbial population. Furthermore, the production of enterotoxins and biogenic amines by *Bacillus* strains is reported (Lee et al., 2019).

Nevertheless, helpful metabolites such as the antimicrobial lipopeptide BSs can be produced by members of the genus. For instance, a miscellaneous of surfactin lipopeptides was isolated from *B*. *subtilis* and *Bacillus amyloliquefaciens* supernatants after 24 h of fermentation on Malaysian fermented food: soybean known as *tempeh* showed a maximum surfactin yield at 84.08 mg L^–1^ and cassava *tapai* the lowest at 26.9 mg L^–1^ (Isa et al., 2020). However, the antimicrobial activity of the soybean BSs was not effective against *S. aureus*, *S. pneumoniae*, *Serratia marcescens*, and *S. typhimurium*, while the BSs from *tapai* inhibited the growth of both Gram-positive and Gram-negative tested bacteria. The most antimicrobial surfactins were produced by the growth on fish sauce *budu* showing a great inhibition halo against *B. cereus* (MIC 10 mg L^–1^) and *S. pneumoniae* (MIC 25 mg L^–1^) and a moderate one against *Listeria monocytogenes* (MIC 25 mg L^–1^), *S. aureus* (MIC 25 mg L^–1^), *K. pneumoniae* (MIC 25 mg L^–1^), and *S. marcescens* (MIC 50 mg L^–1^). Interestingly, Isa et al. (2020) confirmed the surfactin-producing ability of the strains through the detection of the *sfp* marker gene as well as the genus affiliation.

Ultimately, Schlusselhuber et al. (2018) reported *Pseudomonas* strain UCMA 17988, isolated from raw cow milk, for its ability to produce lipopeptide BS, although *Pseudomonas* spp. are famous for rhamnolipid production (Pornsunthorntawee et al., 2010). The maximum yield of 47.6 mg L^–1^ was obtained after 4 days of cultivation. Interestingly, four molecules were identified differing at 14 Da, which suggested the presence of several lipopeptide isoforms. The hypothesis was confirmed by analyzing the differences due to the fatty acid chain: the major isoform was at 1,409 *m*/*z*, and the three other isoforms were detected at 1,381, 1,395, and 1,423 *m*/*z*. Therefore, the lipopeptide BSs were called “milksin” A, B, C, and D. The antimicrobial activity of the major isoform was observed against *S. aureus* CIP 53.154 with MIC of 0.5 mg ml^–1^, against *L. monocytogenes* WSLC 1685, and *Salmonella enterica* Newport CIP 105629 with MIC of 1 mg ml^–1^. Also, the antifungal activity was observed against strains representative of fungal groups: *Mucor hiemalis* CBS 201.65, *Aspergillus niger* CMPG 814, and *Cladosporium herbarum* CMPG 38 showed a major MIC of 20 mg ml^–1^, and *Penicillium expansum* CMPG 136 showed a MIC equal to 20 mg ml^–1^.

#### Glycolipid Biosurfactants

Glycolipids are complex molecules composed of a carbohydrate moiety and a lipid fraction. Although *Pseudomonas* spp. are the most prominent strains reported in the literature as glycolipid BS producers, also Gram-positive bacteria provide the same type of compounds that are released in the environment, for example, microorganisms grouped in the *Lactobacillus* genus.

*Lactobacillus acidophilus* NCIM 2903 is reported to produce a glycolipid type BS in 72 h of fermentation (obtaining 1.5 g L^–1^) (Satpute et al., 2018). Indeed, its chemical characterization revealed the following principal functional groups: hydrocarbon, OH stretching, ester bonds, and sugars. The CMC was 625 μg ml^–1^, which corresponds to a reduction of the ST from 72 to 27 mN m^–1^. At the CMC value, the glycolipid inhibited 87% growth of *S. aureus* NCIM, 85% of *P. aeruginosa* MTCC 2297, 82% of *B. subtilis* MTCC 2423, 80% of *E. coli* NCIM 2065, 70% of *P. putida* MTCC 2467, and *P. vulgaris* of NCIM 2027. Satpute et al. (2018) utilized an innovative approach mimicking the biofilm microenvironment through microfluidic strategies to evaluate the antibiofilm property that showed no biofilm in the presence of the BS.

Also, *Lactobacillus helveticus* M5, isolated from yogurt, releases a glycolipid, characterized by a cycle aliphatic structure of the lipidic moiety when cultivated on lactose (5.5 g L^–1^ yield in 120 h) (Kadhum and Haydar, 2020). It displayed an E_24_ of 75.3% and a reduction of the ST until 33.2 mN m^–1^. Its antimicrobial bioactivity was prevalently against Gram-positive bacteria than Gram-negative bacteria, showing an inhibition halo ranging from 15 to 31 mm against *S. aureus* and from 12 to 29 mm against *P. aeruginosa* at a concentration of between 20 and 100 mg ml^–1^. Thus, the authors speculated that the glycolipid could interfere with the peptidoglycan layer of the Gram-positive bacteria, leading to dysfunctions of the cell wall. Besides, the glycolipid acted as an antiadhesive agent at a concentration of 50 mg ml^–1^, inhibiting 78% and 74.5% of the adhesion of *S*. *aureus* and *P*. *aeruginosa*, respectively.

Among the gut commensal bacteria, *Enterobacter cloacae* B14 produced a glycolipid-like molecule releasing 39.8 mg BS (g cell dry weight)^–1^ when yeast extract is used as a substrate. Its antimicrobial action was more pronounced against Gram-positive bacteria (inhibition haloes 20.7–26.7 mm against *B*. *cereus*, *B*. *subtilis*, and *S*. *aureus*) with respect to the Gram-negative bacteria (9.7–17 mm against *E*. *coli*, *P*. *aeruginosa*, and *S*. *marcescens*). Interestingly, the BS was more effective than the commonly used antibiotic tetracycline against *B*. *subtilis* (respectively 22 vs. 20 mm of growth inhibition), and the BS inhibited the growth of the tetracycline-resistant strain *S*. *marcescens* (Ekprasert et al., 2020).

As already mentioned, *P*. *aeruginosa* is the most studied bacterium for rhamnolipid production. These molecules are formed by a rhamnose moiety linked to an aliphatic variable chain with a BS property. Different rhamnolipids exhibit an antimicrobial activity, such as the ones released by *P. aeruginosa* CR1 (Sood et al., 2020; Wahib et al., 2020).

*Pseudomonas aeruginosa* CR1 BS showed considerable antimicrobial and emulsification activities; indeed, the E_24_ was 53%, and ST decreased until 35 mN m^–1^ (Sood et al., 2020). It was recovered after the strain grew on both Luria Bertani (LB) broth supplemented with glycerol and basal medium enriched with rice bran oil, showing a maximum production of 10 g L^–1^. The chemical analyses revealed that *P. aeruginosa* strain CR1 produced only mono-rhamnolipids and that no di-rhamnolipids were detected. These data were confirmed by genome analyses showing the lack of *rhlC* gene coding for the rhamnosyl transferase responsible for di-rhamnolipid synthesis (Figure 2C and Table 1).

Wahib et al. (2020) evaluated *P*. *aeruginosa* strain, isolated from a clinical source, for its capacity to release 20.04 g L^–1^ of antimicrobial BS when grown on glycerol medium. The BS was characterized as a mixture of mono- and di-rhamnolipids with E_24_ of 88.18%. Interestingly, at a concentration of 0.5 or 1 g ml^–1^, its rhamnolipids could inhibit *E*. *coli*, *K*. *pneumoniae*, and *S*. *aureus* growth, showing the maximum antimicrobial effect against *S*. *aureus*.

#### Glycoprotein Biosurfactants

Intriguingly, from literature retrieval, glycoproteins with antimicrobial and BS features are produced only by the *Lactobacillus* genus. Mouafo et al. (2018) investigated the potential of three *Lactobacillus* strains (*Lactobacillus delbrueckii* N2, *Lactobacillus cellobiosus* TM1, and *L*. *plantarum* G88) to produce BSs during growth on sugar cane molasses or glycerol. Their yields ranged between 2.43 and 3.03 g L^–1^ on sugar cane molasses (with E_24_ ranging between 49.89 and 81%) and from 2.32 to 2.82 g L^–1^ on glycerol (with E_24_ ranging from 41.81 to 61.81%). The molecules produced from the growth on glycerol were composed of a bigger fraction of lipids with respect to the BS obtained on sugar cane molasses. This suggested that lactobacilli could direct the glycerol in the lipolytic pathway and gluconeogenesis, consequently generating more lipids. The growth of *L*. *cellobiosus* TM1 and *L*. *delbrueckii* N2 on sugar cane molasses led to producing glycoproteins without a lipid fraction. The measured protein and sugar content were, respectively, 52.93 g/100 g MS and 27.10 g/100 g MS for *L*. *cellobiosus* TM1-BS, and 63.64 g/100 g MS and 51.13 g/100 g MS for *L*. *delbrueckii* N2-BS. Since the presence of sugars is independent of the carbon source (sugar cane molasses or glycerol), the authors speculated that the hydrophilic substrates were broken down in glycolytic pathway intermediates, such as glucose-6-phosphate, which is the precursor carbohydrate found in the BS composition. The antimicrobial effect indicated that Gram-positive bacteria were more sensitive than Gram-negative. As an example, *Bacillus* sp. BC1 growth was the most affected by the action of *L*. *delbrueckii* N2 glycolipid BS showing 57.5 mm of inhibition zone.

#### Glycolipoprotein Biosurfactants

Likewise, the production of glycolipoprotein BS was recorded only from two *Lactobacillus* strains, *L. plantarum* G88 and *Lactobacillus paracasei* subsp. *tolerans* N2 (Hippolyte et al., 2018; Mouafo et al., 2018), although these complex molecules are often cell-bound because of their big dimensions (see the section “Cell-Associated Biosurfactants”).

Briefly, *L. plantarum* G88 growth on sugar cane molasses produced a molecule characterized by 8.96 g/100 g MS proteins, 51.13 g/100 g MS sugars, and 39.60 g/100 g MS lipids (Mouafo et al., 2018). Distinguishing an antimicrobial activity from that of *E*. *coli* E6, *P*. *putida* PSJ1 and PSV1, and *Salmonella* sp. SL2 was evidenced by the diameter of their inhibition haloes of 32.00, 32.00, 32.00, and 41 mm, respectively.

Curiously, Hippolyte et al. (2018) exploited *L*. *paracasei* subsp. *tolerans* N2’s ability to release bioactive compounds during growth on sugar cane molasses to evaluate the optimization of the production of an antimicrobial BS through a mathematical model. The model outputs were the predicted production yield and two values indicating the BS properties: the diameter of growth inhibition, a measure of the antimicrobial potential, and the ST related to the surfactant effect. After the fermentation under the optimal conditions (temperature between 33°C and 34°C, sugar cane molasses concentration ranging from 5.49 to 6.35%), they obtained an active BS with an experimental ST around 37.02 mN m^–1^, which was comparable with the predicted value (36.65 mN m^–1^). The best glycolipoprotein production conditions for the highest antimicrobial activity comprised the lowest percentages of molasses (5.49%) and the lowest temperature (33°C). The measured inhibition halo against *P. putida* PSJ1 was 63.89 mm, which was comparable with the predicted one (62.07 mm). Then, the antimicrobial activity was assessed against other bacteria: *P*. *aeruginosa* PSB2, *Salmonella* sp. SL2, *E*. *coli* MTCC 118, *Bacillus* sp. BC1, and *S*. *aureus* STP1. *S*. *aureus* and *Bacillus* were the most sensitive bacteria to the glycolipoprotein with a MIC of 3.2 mg ml^–1^, while *Salmonella* and *E*. *coli* were the less sensitive with a MIC of 12.80 mg ml^–1^. Subsequently, a partial chemical characterization revealed that the main constituents were proteins, sugars, and lipids (63.64 g/100 g DM, 35.26 g/100 g DM, and 1.10 g/100 g DM, respectively), suggesting a glycolipoproteins nature.

#### Other Cell-Released Biosurfactants

Some antimicrobial BSs related to bacteria associated with human health were described for their bioactive properties without an exhaustive chemical elucidation; or in few cases, the assembled chemical features make them part of new BS categories.

Although not characterized in-depth, the following examples showed the importance of BS properties for clinical, health-related, and nutrition problems and future applicative developments.

Foschi et al. (2017) focused the attention on the anti-gonococcal potential of *Lactobacillus* strains isolated from healthy premenopausal women. They principally belong to three different species: *Lactobacillus crispatus*, *L*. *gasseri*, and *Lactobacillus vaginalis* among which *L*. *crispatus* strains showed the best anti-*Neisseria gonorrhea* effect. In fact, their supernatant was able to eradicate *N*. *gonorrhea* viability after 7 and 60 min, while *L*. *crispatus* and *L*. *gasseri* species were capable only after 60 min. The most effective was produced by *L*. *crispatus* BC1, also possessing a potent BS property. The characterization of the molecules released in the supernatants indicated that their molecular weight was more than 10 kDa.

The BS extracted from *Pseudomonas synxantha* NAK1 stands out for its interesting biomedical application (Mukherjee et al., 2014). Indeed, the strain, isolated from *Mycobacterium smegmatis* plate, inhibits the *Mycobacterium* growth, which is a non-pathogenic bacterium model for the study of tuberculosis caused by *Mycobacterium tuberculosis* (Yamada et al., 2018). *P. synxantha* NAK1 cultivation generated metabolites that were preliminarily characterized as a 15-carbon aliphatic chain with intermediate oxygen and a terminal allyl bond with surfactant properties. The antimicrobial potential against other bacteria was thoroughly elucidated. The activity was very low against *E*. *coli* DH5α and *P*. *aeruginosa* AKS9 (MIC 200 μg ml^–1^); moderate against *B*. *subtilis*, *Shigella sonnei* NK4010, and *S*. *typhimurium* B10827 (MIC 100 μg ml^–1^); high against *S*. *aureus* ATCC 25923, *M*. *tuberculosis* H_37_Rv, and BGC (MIC 50 μg ml^–1^); and, finally, very high against two *M*. *tuberculosis* strains (mc^2^155 and H_37_Ra, MIC 25 μg ml^–1^). Therefore, this kind of secondary metabolite produced by *P*. *synxantha* NAK1 could be useful as an anti-tubercular agent against the mycobacteria pathogens.

Within the Proteobacteria phylum, other *Pseudomonas* strains revealed promising antimicrobial BSs. *P*. *aeruginosa* ATCC 10145 provides up to 1 g L^–1^ of BS, characterized by an ST lowering capacity of 40 mN m^–1^ (from 72 to 32 mN m^–1^). The BS has also antimicrobial and antifungal activities showing an effect against *Sarcina lutea*, *Micrococcus luteus*, and *Bacillus pumilus*; and among the fungi, the effect was against *Penicillium chrysogenum* and *C. albicans* (El-Sheshtawy and Doheim, 2014).

Among the first paper published regarding the *Lactobacillus* genus within the considered decade (2010–2020), Gudina et al. (2010) described a BS extracted from *L*. *paracasei* ssp. *paracasei* A20, which was isolated from Portuguese dairy plant. The extracted molecule was tested against 18 microorganisms, including species associated with the oral cavity, pathogenic bacteria, yeasts, and skin-associated pathogenic fungi. The antimicrobial potential was observed vs. all the strains, and the growth inhibition was observed for around 67% of the microorganisms at 50 mg ml^–1^. Only the cariogenic *Streptococcus mutans* strains NS and HG985, *P*. *aeruginosa*, the yeast *Malassezia* sp., and the fungi *Trichophyton mentagrophytes* and *Trichophyton rubrum* were non-sensible to the BS molecule. Regarding the antiadhesive capacity, a BS concentration of 50 mg ml^–1^ inhibited the non-pathogenic *Lactobacillus reuteri* and *L*. *casei* of 77.6–78.8% and 56.5–63.8%, respectively.

The work of Gomaa (2013) described 10 *Lactobacillus* strains isolated from Egyptian dairy products among which *L*. *paracasei* produced a BS with an antimicrobial activity against *C*. *albicans*, *S*. *aureus*, and *Staphylococcus epidermidis.* Therefore, the authors compared this capacity with that of *L*. *paracasei* A20. Results showed that the novel extracted BSs demonstrated more potent antiadhesive compounds with respect to A20 strain. However, the best antiadhesive potential was attributable to *Lactobacillus fermentum* bioactive molecule (84.69% of inhibition) (Gomaa, 2013).

Other BS molecules were produced by strains isolated from food matrices. Two *L*. *plantarum* strains, called L26 and L35, and *L*. *brevis* strain L61, isolated from a Romanian traditional fermented food, produced BSs with an antimicrobial effect only against *E*. *coli* (Cornea et al., 2016).

Another example of BS from food derivatives is the screening of BS-producing capacity of bacteria isolated from dairy products, breast milk, fermented shrimps, and fruits (Abdalsadiq and Zaiton, 2018). Among 160 bacteria and 70 randomly selected to test the BS activity, only 20 cell-free supernatants were positive to drop collapse test and oil spreading assay. Furthermore, only six of the isolates were able to reduce the water-surface tension, leading to an average reduction from 72.22 to 37.21 mN m^–1^. The antibacterial activity was evidenced only for nine cell-free supernatants. Among them, the isolate *L*. *acidophilus* Fm1 was the most effective because it could inhibit the growth of *Pseudomonas fluorescence* (33.4 mm of zone inhibition), *S*. *typhimurium* (30.4 mm), *P*. *aeruginosa* ATCC 2785 (29.7 mm), *P*. *aeruginosa* 14T28 (25.5 mm), and *E*. *coli* (20.2 mm) (Abdalsadiq and Zaiton, 2018).

Moreover, among the four *L*. *plantarum* strains (Is2, Is9, Is12, and Is13) isolated from plantain wine (*Mbamvu*, or banana wine), a typical African fermented beverage, one isolate showed interesting BS and antimicrobial features. It was able to strongly inhibit the growth of selected pathogens, such as *E*. *coli* (3.3 cm of growth inhibition halo), *Shigella flexneri* (4.2 cm), *Salmonella* sp. (3.3 cm), *P*. *aeruginosa* (3.5 cm), and *S*. *aureus* (4 cm) (Moukala et al., 2019).

The last research studies represent an important description of the properties of bacterial bioactive compounds related to food and beverage fermented matrices as beneficial products for people’s health and ultimately to raise knowledge about nutritional issues (Parthasarathi and Subha, 2018).

## Application of Bacteria-Derived Biosurfactants With Antimicrobial Activity

The attention of the scientific community on BS antimicrobial compounds is rapidly growing because of their intrinsic characteristics of BSs and antimicrobial agents, the interest towards the producer strains, and their low organismal and environmental impact.

The intrinsic surface-active capacity of these compounds can be evaluated by drop-collapsing method and oil displacement tests and quantified by a tensiometer that measures the reduction of water-surface tension (Walter et al., 2010). In general, the most efficient surfactant molecules are those able to reduce the water-surface tension from 72 to around 30 mN m^–1^ compared with a standard at a defined condition (Saimmai et al., 2020). One of the most powerful antimicrobial BSs from bacteria associated with human health is the glycolipid from *Lactobacillus acidophilus* NCIM 2903 since it reduced the ST to 27 mN m^–1^ (Satpute et al., 2018). Another effective BS is produced by *Bacillus subtilis* VSG4, which showed a minimum ST value of 27.2 mN m^–1^ at pH 7 (Giri et al., 2019). BSs produced by members of *Lactobacillus* genus can be also considered as effective surfactants compared with synthetic ones (Santos et al., 2016). For instance, the BS released by *Lactobacillus paracasei* subsp. *tolerans* N2 lowered the ST to 37.85 mN m^–1^ (Hippolyte et al., 2018), while the glycolipoproteins from *Lactobacillus jensenii* P_6A_ and *Lactobacillus gasseri* P_65_ reduced the water-surface tension to 43.2 and 42.5 mN m^–1^, respectively (Morais et al., 2017).

Considering the other bioactive functions of these compounds deriving from bacteria associated with human health, some authors suggest that the antimicrobial BSs produced by *Lactobacillus* spp. could be employed in the prevention or treatment of hospital-acquired infections. Indeed, these new antimicrobial BS agents showed an antagonist effect against bacteria causing infections and diseases in the urinary, vaginal, and GI tracts, as well as in the skin (Gudina et al., 2010); a very low cytotoxic effect on human lung epithelial cells; and antimicrobial, antiadhesive, and antibiofilm capacity against clinical MDR strains (Sambanthamoorthy et al., 2014). Additionally, they can be used on various surfaces of biomedical devices as antimicrobial, antiadhesive, and antibiofilm agents (Satpute et al., 2019; Kadhum and Haydar, 2020) or for controlling bacterial overgrowth in the food and nutraceutical industry (Cornea et al., 2016; Hippolyte et al., 2018; Mouafo et al., 2018; Emmanuel et al., 2019; Moukala et al., 2019; Isa et al., 2020; Supplementary Table 1).

Consequently, the number of potential applications in different fields, especially in biomedical, food safety, and nutraceutical sectors, is currently increasing.

Moreover, nowadays, the interest in greenways of producing add-value compounds is rapidly growing, in this specific case, the attention is towards BSs with an antimicrobial activity.

Therefore, it is fundamental to underline the importance of the valorization of by-products, residues, and wastes through biological processes to avoid the loss of other useful resources for new business models (Hippolyte et al., 2018; Mouafo et al., 2018; Singh et al., 2018; Deseo et al., 2020).

Biosurfactants derived from human-associated bacteria that utilize by-products or cheap substrates could have a great impact on biotechnological and industrial levels. For instance, various members of the *Lactobacillus* genus are able to produce valuable antimicrobial BS molecules during the fermentation of sugar cane molasses, which are products with generally recognized as safe (GRAS) status of the steam process of the sugar cane mill industry (Hippolyte et al., 2018; Mouafo et al., 2018; Deseo et al., 2020). Consequently, the need for more research studies and in-depth investigations on novel bioactive compounds with a high rate of production for other potential applications is still high.

### Biomedical Applications

Nowadays, the resistance to antimicrobial substances represents a big challenge to face, above all in the hospital environment. Indeed, the use of the BSs as alternatives to conventional antibiotics is a promising answer to this issue that can be developed by *Lactobacillus* BSs (Gudina et al., 2010; Hippolyte et al., 2018). These bioactive compounds are considerably efficient against potential pathogens responsible for diseases and infections in the urinary, vaginal, and GI tracts, as well as in the skin. The BSs produced by *L*. *jensenii* P_6A_ and *L*. *gasseri* P_65_ can have a role as antibiotic agents for the vaginal compartment, because of their significant antimicrobial activities against *Escherichia coli* and *Candida albicans* and their antiadhesive potential against *E*. *coli*, *Staphylococcus saprophyticus*, and *Enterobacter aerogenes* (Morais et al., 2017).

The need for “antibiotic-free” strategies vs. genital diseases, such as gonorrhea, can be accomplished by safe bioactive compounds. For example, Foschi et al. (2017) suggested that the BS isolated from *Lactobacillus crispatus* BC1 is capable of killing *Neisseria gonorrhoeae* after a short contact period. This novel isolated compound could be used in the prevention of *N*. *gonorrhoeae* infections in women since the disturbing pathogen had developed antimicrobial resistance.

In the field of oral diseases and their prevention, the BS-LBI and BS-LZ9 BSs from *Lactobacillus casei* LBI could be employed for their antibiofilm capacity that was demonstrated against two *Staphylococcus aureus* strains isolated from the oral cavity of Tunisian patients. Moreover, the BS molecules tested on human epithelial cell line HEp-2 showed an antiproliferative effect (Merghni et al., 2017).

Of particular interest is the BS coating agents of surfaces, such as catheters or other instruments for biomedical support. Satpute et al. (2019) extracted a glycolipoprotein-type BS from the cell surface of *L*. *acidophilus* NCIM 2903 that showed antibiofilm and antiadhesive activities on polydimethylsiloxane-based contact lens surfaces. This kind of study deals with the problem of the failure of implants due to the colonization of biofilm-forming microorganisms, often resistant to antibiotics. In this context, the glycolipid released by *Lactobacillus helveticus* M5 with the antimicrobial and antiadhesive actions against *Pseudomonas aeruginosa* and *S*. *aureus* could be also very useful as a coating agent (Kadhum and Haydar, 2020).

In line with these discoveries, the antibiofilm, antiadhesive, and antimicrobial BSs extracted from *L*. *jensenii* and *Lactobacillus rhamnosus* support the employment of BSs on abiotic surfaces as a medical coating instrument to combat microbial colonization. It could be also speculated to use them in a topical application since both the molecules resulted in low cytotoxicity on human A549 lung epithelial cells (Sambanthamoorthy et al., 2014).

The studies conducted by Gomaa (2013), Mukherjee et al. (2014), Abdalsadiq et al. (2018), Abdalsadiq and Zaiton (2018), Satpute et al. (2018), Clements et al. (2019a), Emmanuel et al. (2019), Ghasemi et al. (2019), and Giri et al. (2019) provided new diverse antimicrobial BSs deriving from different bacteria with possible employment in the biomedical field. Their relevance is linked to the field as adjuvants in immunology, drug delivery system, therapeutic agents, gene deliveries, antimicrobial, antiadhesive, and antibiofilm agents (Saimmai et al., 2020).

### Application in Food and Nutraceutical Industries

In the field of the food industry, the most important factor is the quality of the products, which is strictly connected to their provenience, maintenance, and storage and the product safety for the health of the consumers (Nalini et al., 2020). Therefore, texture, consistency, aroma, taste, and safety had a role in the perception of the food and the assignment of excellence. In this context, surfactants deriving from microorganisms are more advantageous than chemical ones, because they are less toxic, biodegradable, and eco-friendly (Nalini et al., 2020).

Among the producer strains, lactobacilli are interesting because they usually have the GRAS status, and they are also naturally present in the food products.

One of the principal applications of BSs in the food industry is as bio-emulsifiers. The emulsifying properties of BSs from *Lactobacillus* strains were evaluated and exploited against edible oils such as sunflower and olive oil (Cornea et al., 2016; Emmanuel et al., 2019). Besides, the antimicrobial activity of *Lactobacillus* sp. strain from homemade curd BS was effective against *E*. c*oli* (Emmanuel et al., 2019), and one of the lactobacilli derived from Romanian traditional fermented food BSs was effective also against *Bacillus cereus*, as a food pathogen (Cornea et al., 2016).

In the same context, Hippolyte et al. (2018) and Mouafo et al. (2018) isolated, respectively, *L*. *paracasei* subsp. *tolerans* N2 (capable of producing an antimicrobial BS from traditional fermented milk known in Cameroon as “*pendidam*”) and *Lactobacillus delbrueckii* N2, *Lactobacillus cellobiosus* TM1, and *Lactobacillus plantarum* G88 for their fermenting ability of sugar cane molasses and the production of antimicrobial BSs that generate stable emulsions for at least 72 h at room temperature. These properties lead to the hypothesis that the molecules can form and stabilize emulsions and could be suitable as bio-preservatives.

It is important to underline that it is preferable to use non-pathogenic organisms in a bioprocess, such as in the case of the antimicrobial BS production from *Pediococcus dextrinicus* strain SHU1593 (Ghasemi et al., 2019).

Moreover, the isolated bioactive molecule should be defined as safe to utilize as antimicrobial agents in products that come in contact with humans, such as food and beverage products. For this reason, it is fundamental to verify this requirement, for instance, through the evaluation of the viability of a cell line in the presence of a bioactive compound under analysis (Basit et al., 2018). Another important requirement for bioactive compounds with surfactant and emulsification features is the antimicrobial activity for the biocontrol of pathogenic and food spoilage bacteria as suggested by Giri et al. (2019). Indeed, *B*. *subtilis* VSG4 and *Bacillus licheniformis* VS16 BSs showed stable emulsification and appropriate ST values in pH values, respectively, ranging from 4 to 10 and from 5 to 9 and in the presence of different temperatures, from 20°C to 90/100°C for 30 min. Besides, they exert their antimicrobial potential against both Gram-positive and Gram-negative bacteria (Giri et al., 2019).

Intriguingly, Moukala et al. (2019) and Isa et al. (2020) raised the attention on the local fermented food beverages as a source of bioactive molecules together with the intrinsic bioactive substances such as polyphenols, flavonoids, and carotenoids. The BSs produced by *L*. *plantarum* strains and *P*. *aeruginosa* strain, isolated, respectively, from banana wine and Malaysian fermented foods, could have a role not only in the food industry but also in the health state of the local population. BSs can be also important molecules for the nutraceutical industry to stabilize the formulation thanks to the emulsification and stabilization properties, and the antiadhesive and antimicrobial capacity.

## Conclusion and Future Perspectives

Bacteria associated with human health are capable of producing antimicrobial BSs with great biomedical potential, useful in the food industry and generally beneficial for human health. For example, the employment of the antimicrobial and antibiofilm molecules could prevent hospital-acquired infections. Furthermore, molecules with antibiofilm properties could be utilized in the eradication and preservation of urogenital infections in addition to or as replacements of conventional antibiotics.

In the field of the BS industry, the search for novel strains able to ferment by-products or able to use renewable/cheaper substrates is very important and constantly increasing. Moreover, the application of molecules derived from GRAS bacteria that are already defined as safe for contact with human bodies is fruitful for the food industry.

Generally, some bioactive compounds can be employed for environmental application, thus improving the ecosystem from which our food derives and the habitat we are in contact with.

An important goal that still needs to be reached is the elucidation of the chemical features of the already extracted but not yet characterized BSs, to enhance the opportunities for possible therapeutic approaches. Furthermore, deeper knowledge about the encoding genes for the BS production or insight into the mechanisms involved in the production process could be interesting for the prediction of this capability, to better set up a development and the scale-up of possible industrial projects.

## Author Contributions

AD conceived the review and developed the whole manuscript by writing the different text parts. JZ wrote the section “Prediction of Biosurfactant With an Antimicrobial Activity by an “*-omic*” Approach” and revised the whole manuscript by providing useful suggestions. PD helped in shaping the manuscript. All the authors provided critical feedback and contributed to the final manuscript.

## Conflict of Interest

The authors declare that the research was conducted in the absence of any commercial or financial relationships that could be construed as a potential conflict of interest.
