# Supplementary material for: Recent Trends on Biosurfactants With Antimicrobial Activity Produced by Bacteria Associated With Human Health: Different Perspectives on Their Properties, Challenges, and Potential Applications
Source: Front Microbiol. 2021 Apr 23;12:655150. doi: 10.3389/fmicb.2021.655150 (PMC8104271; doi:10.3389/fmicb.2021.655150)
Supplement: Supplementary file 1 [file Table_1.docx]

**Table S1** Description of biosurfactants with antimicrobial activity from bacteria associated with human health: types, main characteristics, production methods along with their potential applications

| **Strain** | **Isolation site** | **Habitat** | **BS type** | **BS characteristics** | **Production and characterization** | **Bioactive function** | **Potential application** | **Reference** |
| --- | --- | --- | --- | --- | --- | --- | --- | --- |
| *Bacillus amyloliquefaciens* and *Bacillus thurigirnsis* | Puba (regional fermentation product from cassava) | *B. amyloquefaciens*: agriculture, aquaculture, and hydroponics. *B. thurigiensis*: soil | Lipopeptide | Cell-free iturin A (m/z 1079) and surfactin A and B (m/z 1030) | Production after 48 h. The supernatant was adjusted to pH 2 with HCl and precipitated at 4°C for 16 h, then extracted with dichloromethane. Alternatively, extracted with ¼ volume of n-butanol. Characterized with RP-HPLC and MALDI-TOF analysis | Antimicrobial activity of *B. amyloliquefaciens* BS against *B. amyloliquefaciens* LBM 5006 and ATCC 23350; *B. cereus* ATCC 14579 (also inhibited by *B. thurigirnsis* BS), A-1, B-2, and D1 (also inhibited by *B. thurigirnsis* BS); *B. subtilis* ATCC 6633, DSM 3258, ATCC 21228, and ATCC 7971; *C. fimi* NTCS 7547; *L. acidophilus* ATCC 4356; *L. murinus* L2; *L. monocytogenes* ATCC 6477, ATCC 15113, ATCC 19112, and ATCC 19115; *L. innocua*; *S. aureus* ATCC 25923; *S. haemolyticus*; *S. saprophyticus*, and *E. aerogenes*; antifungal against *A. flavus* (also inhibited by *B. thurigirnsis* BS); *A. fumigatus*; *A. niger* (also inhibited by *B. thurigirnsis* BS); *F. oxysporum* f. sp. *lycopersici*; and *C. tropicalis* | - | Perez et al., 2017 |
| *Bacillus subtilis* and *Bacillus amyloliquefaciens* | Malaysian fermented food: *B. subtilis* from *budu* (fish sauce) and *tempeh* (fermented soybeans); *B. amyloliquefaciens* from *cincalok* (fermented small shrimps) and *tapai ubi kayu* (fermented cassava) | *B. subtilis*: Soil and human gastrointestinal tract *B. amyloquefaciens*: agriculture, aquaculture, and hydroponics | Lipopeptide | Cell-free surfactin | Production after 24 h. Supernatant was acidified. Then purified through HPLC. Characterized by LCMS analysis | Antimicrobial against *B. cereus* ATCC 13061, *L. monocytogenes* ATCC 13932, *S. aureus* ATCC 25923, *S. pneumoniae* ATCC 6303, S. *typhimurium*ATCC 13311, *S. marcescens* ATCC 14756, and *K. pneumoniae* ATCC 10031; hemolytic activity | Potential for curbing the ill effects arising from pathogenic bacteria present in food | Isa et al., 2020 |
| *Bacillus subtilis* VSG4 and *Bacillus licheniformis* VS16 | Kitchen waste dumping site and household food waste samples | *B. subtilis*: Soil and human gastrointestinal tract *B. licheniformis*: soil | Lipopeptide | Cell-free BS-VSG4 and BS-VS16 | Production after 96 h for B. subtilis, while production after 72 h for B. licheniformis. The supernatant was precipitated by acidifying to pH 2 with 6N HCl | Antimicrobial against *S. aureus* ATCC 29523, *B. cereus* MTCC 7190, *E. coli* MTCC 65, *S. tiphymurium* ATCC 19430, and *V. parahaemolyticus* MTCC 735; anti-adhesive against *S. aureus* ATCC 29523, *S. tiphymurium* ATCC 19430, and *B. cereus* ATCC 11778; anti-biofilm against *S. typhimurium*, *S. aureus*, and *E. coli;* antioxidant activity | Potential use in the biomedical and food industry | Giri et al., 2019 |
| *Enterobacter cloacae* B14 | Culture Collection Center at the Department of Microbiology, Faculty of Science, Khon Kaen University | Human gastrointestinal tract | Glycolipid | Cell-free glycolipid | Production after 96 h. The pH of the supernatant was adjusted to 2.0 with 6N HCl. Then kept at 4°C overnight. Extracted with chloroform. Characterized by TLC and FTIR analysis | Antimicrobial against *E. coli*, *P. aeruginosa*, *S. marcescens*, *B. cereus*, *B. subtilis*, and *S. aureus* | Potential application for bioremediation and the production of antimicrobial products | Ekprasert et al., 2020 |
| *Lactobacillus acidophilus* and *Lactobacillus pentosus* | - | *L. acidophilus*: human and animal gastrointestinal tract and mouth. *L. pentosus*: olive | Lipopeptide and glycolipid fractions | Lipopeptide and glycolipid fractions cell-free biosurfactants | Extraction with chloroform and methanol; purified by column chromatography; characterized by TLC | Antimicrobial, anti-adhesion and antibiofilm activity against *P. mirabilis*, *S. aureus*, *S. pneumonia*, *K. pneumoniae*; antifungal against *C. albicans* | Controlling biofilm development and influence the adhesion ability of pathogens | Abdalsadiq et al., 2018 |
| *Lactobacillus acidophilus* NCIM 2903 | National Collection of Industrial Microorganisms (NCIM) | Human and animal gastrointestinal tract and mouth | Glycolipid | Cell-free glycolipid (MW=45 kDa) | Production after 72 h. The supernatants were acidified to pH 2 with 5N HCl, stored overnight at 4°C, and extracted with ethyl acetate/methanol (4:1) mixture; purification through chromatographic technique; the ionic character of the biosurfactant was determined through agar double diffusion assay; chemical characterization was developed with TLC and FTIR analysis | Antimicrobial against *S. aureus* NCIM 2079, *P. aeruginosa* MTCC 2297, *B. subtilis* MTCC 2423, *E. coli* NCIM 2065, *P. putida* MTCC 2467, and *P. vulgaris* NCIM 2027; antibiofilm and anti-adherent against *B. subtilis* MTCC 2423, and *P. vulgaris* 2027 | Role in the prolongation of the life of the biomaterials; potential antiadhesive application on different surfaces of biomedical devices | Satpute et al., 2018; Satpute et al., 2019 |
| *Lactobacillus acidophilus, Lactobacillus pentosus,*and *Lactobacillus fermentum* | Dairy products, breast milk, fermented shrimp, and fruits samples in Malaysia | *L. acidophilus*: human and animal gastrointestinal tract and mouth; *L. pentosus:* olive; *L. fermentum*: dental caries lesions. It is also commonly found in fermenting animal and plant material, and sourdough | - | Uncharacterized cell-free biosurfactants | Production after 72 h. For the extraction, the pH of cell free supernatant was adjusted to 6.2 with 1N NaOH | Antimicrobial against *P. florescence*, *P. aeruginosa* ATCC 2785 and 14T28, *E. coli* and *S. typhimurium*; haemolytic activity | Enhancing the antimicrobial properties and medicinal benefit | Abdalsadiq and Zaiton, 2018 |
| *Lactobacillus casei* LZ9*,* and *Lactobacillus casei* LBI | - | Human urinary tract and mouth | - | Uncharacterized cell-associated biosurfactant | Production after 48 h. The cell pellets were collected by centrifugation, washed and resuspended. The biosurfactants were released | Antimicrobial, anti-adhesive and antibiofilm against *S. aureus* ATCC 6538, 9P and 29P; scavenging activity; antiproliferative effect on epithelial cell line (HEp-2) | Natural source for limiting or preventing oral infections and diseases | Merghni et al., 2017 |
| *Lactobacillus crispatus* BC1 | Vaginal swabs of healthy premenopausal Caucasian women | Vagina and human gut | - | Cell-free biosurfactant (MW > 10,000) | Production at a turbidity of 8–10 McF. The cell pellets were collected by centrifugation, washed, and resuspended. The biosurfactants were released | Antimicrobial activity against *N. gonorrhoeae* | Potential probiotic strategy for the prevention of GC infections in women | Foschi et al., 2017 |
| *Lactobacillus delbrueckii* N2, *Lactobacillus cellobiosus* TM1, and *Lactobacillus plantarum* G88 | Ground beef sold in Adamawa region of Cameroon | *L. delbrueckii*: microbiota of the lower reproductive tract of women. *L. cellobiosus* (= *L. fermentum*): dental caries lesions. It is also commonly found in fermenting animal and plant material, and sourdough. *L. plantarum: f*ermented food products as well as anaerobic plant matter. It is also present in saliva. | Glycoprotein, glycolipid, and glycolipoprotein | Cell-free glycoproteins, glycolipids and glycolipoproteins | Production after 72 h. The supernatants were acidified to pH 2 with 6N HCl, stored overnight at 4°C, and extracted three times with equal volume of ethyl acetate/methanol (4:1); the presence of phosphates, total proteins content, total sugars content, and lipid content was estimated using chemical reactions | Antimicrobial against *Bacillus* sp. BC1 and *S. aureus* STP1; antifungal against *C. albicans* LV1 | Application in food industry as emulsifier or biopreservatives | Mouafo et al., 2018 |
| *Lactobacillus helveticus* M5 | Yogurt | Several cheeses | Glycolipid | Cell-associated glycolipid with cycle aliphatic lipid nature of the structures | Production after 120 h. The cell pellet is washed and then resuspended in PBS. After 24 h, the supernatant is extracted with chloroform:methanol (2:1). Characterized by FTIR and GC-MS analysis | Antimicrobial and anti-adhesive against *P. aeruginosa* and *S. aureus* | Possible use in reducing microbial adhesion combating colonization by pathogenic biomedical microorganisms | Kadhum and Haydar, 2020 |
| *Lactobacillus jensenii* 25258 and *Lactobacillus rhamnosus* 7469 | American Type Culture Collection (ATCC) | *L. jensenii*: lower reproductive tract in healthy women. *L. rhamnosus*: healthy female genito-urinary tract and gut | - | Uncharacterized cell-associated biosurfactant | Production after 48 h. The cell pellets were collected by centrifugation, washed and resuspended. The biosurfactants were released | Antimicrobial against *A. baumannii*, *E. coli*, strains UAMS-1 and methicillin resistant *S. aureus*, and *K. pneumoniae*; antibiofilm against *A. baumannii*, *E. coli*, and *S.aureus*; very low toxicity levels against human A549 lung epithelial cells | Possible use as coating agents for medical surfaces or devices to prevent infections caused by microbial colonization | Sambanthamoorthy et al., 2014 |
| *Lactobacillus jensenii* P6A and *Lactobacillus gasseri* P65 | Vaginal fluids of healthy women | *L. jensenii*: lower reproductive tract in healthy women. *L. gasseri*: vaginal flora | Glycolipoprotein | Cell-bound glycolipoproteins (rhamnose as sugar moiety) | Production after 72 h. The cell pellets were collected by centrifugation, washed and resuspended. The biosurfactants were released; protein concentrations, total carbohydrate concentrations, and lipids quantification were developed with chemical reactions; characterized by GC-MS and FTIR analysis | Antimicrobial against *E. coli*, *S. saprophyticus*, *E. aerogenes* and *K. pneumoniae*; antibiofilm against *E. aerogenes* (P6A biosurfactant), *E. coli* and *S. saprophyticus* (P65 biosurfactant); antifungal against *C. albicans* | Possible use as alternative antimicrobial agents in medicine for applications against pathogenic microorganisms that are responsible for infections and diseases in the gastrointestinal and urogenital tracts and the skin | Morais et al., 2017 |
| *Lactobacillus paracasei* ssp. *paracasei* A20 | Portuguese dairy plant | Many human habitats such as human intestinal tracts and mouths; as well as sewages, silages, and dairy products | - | Uncharacterized cell-free biosurfactants | Production after 72 h. For the extraction, cells were harvested by centrifugation, washed and resuspended in phosphate-buffer; then bacterial cells were removed by centrifugation, the supernatant filtered and dialysed | Antimicrobial and anti-adhesive against *L. casei* 36 and 72, *L. reuteri* 104R ML1, *S. mutans* NS and HG985, *S. oralis* J22, *S. sanguis* 12; *E. coli*, *P. aeruginosa*, *S. aureus*, *S. epidermidis*, *S. agalactiae*, *S. pyogenes*; antifungal against *C. albicans*, *Malassezia* sp., *T. mentagrophytes*, *T. rubrum* | Antimicrobial agent in the medical field for applications against microorganisms responsible for diseases and infections in the urinary, vaginal and gastrointestinal tracts, as well as in the skin | Gudina et al., 2010 |
| *Lactobacillus paracasei subsp. tolerans* N2 | Fermented cow milk “pendidam” sold in Ngaoundéré (Cameroon) | Many human habitats such as human intestinal tracts and mouths; as well as sewages, silages, and dairy products | Glycolipoprotein | Cell-free glycolipoprotein | Production at the end of the fermentation. The cell-free supernatant was acidified to pH 2.0 with 6 N HCl, stored overnight at 4°C and extracted three times with ethyl acetate and methanol (4:1); the total protein content, the total sugar content, and the total lipid content were chemically evaluated. Characterized by FTIR analysis | Antimicrobial against *P. aeruginosa* PSB2, *P. putida* PSJ1, *Salmonella* sp. SL2, *E. coli* MTCC 118, *Bacillus* sp. BC1, *S. aureus* STP1 | Application in the medical field and in the food industry | Hippolyte et al., 2018 |
| *Lactobacillus paracasei, Lactobacillus plantarum, Lactobacillus delbrueckii, Lactobacillus acidophilus, Lactobacillus casei, Lactobacillus fermentum, Lactobacillus rhamnosus, Lactobacillus spp., Lactobacillus brevis,* and *Lactobacillus reuteri* | Traditional Egyptian dairy products collected from the Cairo markets | *L*. *paracasei*: Many human habitats such as human intestinal tracts and mouths; as well as sewages, silages, and dairy products. *L. plantarum*: fermented food products as well as anaerobic plant matter. It is also present in saliva. *L. delbrueckii*: microbiota of the lower reproductive tract of women. *L. acidophilus*:  human and animal gastrointestinal tract and mouth. *L. casei*: Human urinary tract and mouth. *L. fermentum*: dental caries lesions. It is also commonly found in fermenting animal and plant material, and sourdough. *L. rhamnosus*: gut microbiota and healthy female genito-urinary tract. *L. brevis*: intestine, vagina, and feces. *L. reuteri*: mammalian gut microbiota. | - | Uncharacterized cell-free biosurfactants | Production after 72 h. The cell-free supernatant was acidified to pH 2 with 6 M HCl, stored overnight at 4°C; extraction three times with chloroform/methanol | Antimicrobial and anti-adhesion against *B. subtilis* 1020 (originally ATCC 6633) and 1250 (originally NCTC 10400), *S. aureus* 1351 (originally NCTC 7447), *P. aeruginosa* 1259 (originally ATCC 10145), *S. typhi* 1350 (originally NCIMB 9331), *P. vulgaris* 1227 (originally ATCC 27973); antifungal activity against *C. albicans* 22 CBS 5703 (originally ATCC 70014) | - | Gomaa, 2013 |
| *Lactobacillus plantarum* (Is2, Is9, Is12 and Is13) | Banana wine (plantain wine (Mbamvu)) | *L. plantarum*: fermented food products as well as anaerobic plant matter. It is also present in saliva | - | Uncharacterized cell-free biosurfactants | Production after 24 h. Supernatant extracted with chloroform (1:1) or with ammonium sulphate (80%) precipitation | Antimicrobial against *S. flexneri*, *Salmonella* spp., *P. aeruginosa*, and *S. aureus* | Agent for the control of local beverages production | Moukala et al., 2019 |
| *Lactobacillus plantarum* L26 and L35, and *Lactobacillus brevis* L61 | Romanian traditional fermented food products | *L. plantarum*: fermented food products as well as anaerobic plant matter. It is also present in saliva. *L. brevis:* intestine, vagina, and feces | - | Uncharacterized cell-free and cell-associated biosurfactants | Production after 24h with maximum at 48 h. For isolation of the cell-bound biosurfactants: centrifuge and washed; for excreted biosurfactants recovery: cell-free supernatants were acidified with 12 N hydrochloric acid to pH 2.0 | Antimicrobial against *E. coli*, *B. cereus*, *S. aureus*; antifungal against *C. parapsilopsis* and *C. mycotoxigenic* | Employment in food industry because of emulsifying properties against edible oils and for antimicrobial properties | Cornea et al., 2016 |
| *Lactobacillus* sp. | home-cured curd | Related to the human body, and food | Lipopeptide | Cell-free lipopeptide with alkene and alkyl groups | Production after 24 h. The pH of cell-free supernatant was adjusted to 2.0 using 6 N HCl. Extraction with equal amount of chloroform: methanol; characterized by TLC and FTIR | Antimicrobial and antibiofilm against *E. coli* | Application in environmental remediation, food industry and in medical uses | Emmanuel et al., 2019 |
| *Pediococcus dextrinicus* SHU1593 (*L. dextrinicus*) | Collection of Shiraz University, Iran | Fermenting vegetables and beverages such as beer and wine | Lipoprotein | Cell-associated lipoprotein | Production after 48 h. The cell pellets were collected by centrifugation, washed, and resuspended. The biosurfactant was then released. The obtained aqueous suspension was acidified with 1M HCl till pH 2. The precipitated biosurfactant was collected by centrifugation. Protein, carbohydrate, and lipid content were assayed chemically and through GC-FID analysis; characterized by FTIR analysis | Antimicrobial against *E. aerogenes*, *E. coli*, and *P. aeruginosa*; anti-adhesive against *B. cereus*, *P. aeruginosa*, and *S. typhimurium* | Possible application in pharmaceutical and food industries | Ghasemi et al., 2019 |
| *Pseudomonas aeruginosa* | Clinical sources | Soil, water, skin, and most man-made environments | Glycolipid | Cell-free rhamnolipid | Production after 120 h. Extracted with organic solvent methods (methanol/chlorofom/acetone 1:1:1). Purified through column chromatography. Characterized by TLC analysis | Antimicrobial against *S. aureus*, *K. pneumoniae,* and *E. coli* | Application as biocontrol agent | Wahib et al., 2020 |
| *Pseudomonas aeruginosa* ATCC 10145 | Microbial resources center (MIRCEN), Faculty of Agriculture, Ain Shams University, Cairo, Egypt | Soil, water, skin, and most man-made environments | - | Uncharacterized cell-free biosurfactants | Production after 96 h. Supernatant acidified till pH2, then extraction with chloroform-methanol (2:1) three times | Antimicrobial against *S. lutea*, *M. luteus*, and *B. pumilus*; antifungal against *P. chrysogenum*, and *C. albicans* | Biocontrol agent | El-Sheshtawy et al., 2014 |
| *Pseudomonas aeruginosa* CR1 | Rhizosphere of chili plantation grown under high chemical inputs | Soil, water, skin, and most man-made environments | Glycolipids | Cell-free mono-ramnolipids (Rha-C10-C10 with 503 m/z, Rha-C10-C8 with 475 m/z, and CH_3_-Rha-C12:2-C10:1 with 539 m/z) and Pyocyanin | Production after 54 h. Supernatant was acidified till pH2, left overnight at 4°C. extraction with ethyl acetate (1:1). Characterized by FTIR, TLC, and LC-MS analysis | Antibiofilm against *B. amyloliquefaciens* CD16, *B. velezensis*, and *B. subtilis* MBGLi97 | - | Sood et al., 2020 |
| *Pseudomonas* sp. UCMA 17988 | Raw cow milk | Soil, water, and plants | Lipopeptide | Cell-free milksin A, B, C, and D (amphisin family) | Production after 96 h. Supernatant extracted three times with ethyl acetate (1:1). Characterized by RT-HPLC, MALDI, and NMR analysis | Antimicrobial against *S. aureus* CIP 53.154; *S. enterica* serotype Newport CIP 105629, Typhimurium LMG 7233, Dublin CIP 7053, Mbandaka CIP 105859, Montevideo CIP 104583; *E. coli* O157:H7 *stx*-C267S and K12 ATCC 1079; *L. monocytogenes* WSLC 1685; *P. aeruginosa* LMG 01242T; *E. faecium* CIP 103014T; antifungal against *A. niger* CMPG 814, *C. herbarum* CMPG 38, *M. hiemalis* CBS 201.65, and *P. expansum* CMPG 136 | - | Schlusselhuber et al., 2018 |
| *Pseudomonas synxantha* | Contaminant on *M. smegmatis* plate | Soil and water (*P. fluorescens* group) | - | Cell-free long-chain aliphatic hydrocarbon with a terminal alyl bond and intermediate electronegative atom biosurfactant (343.13 m/z) | Production after 72 h. Concentrated supernatant extracted with dichloromethane-methanol (4:1). Characterized by NMR, ESI-MS, and FTIR analysis | Antimicrobial against *E. coli* DH5α, *B. subtilis*, *P. aeruginosa* AKS9, *S. aureus* ATCC 25923, *S. sonnei* NK4010, *S. typhimurium* B10827, *M. smegmatis* mc^2^155, *M. tuberculosis* strains H_37_Ra, H_37_Rv and BCG; hemolytic activity | Anti-myco-bacterial agent | Mukherjee et al., 2014 |
| *Serratia marcescens* | Municipal wastewater treatment plants (WWTPs) with samples collected from the influent point, aeration tanks, settling tanks and effluent points; olive oil, wine effluent samples were collected at the points; inlet point, four different compartments of a bioreactor, sludge from one of the bioreactor compartments and an outlet point of an oil refinery treatment plant following the washing or crushing of olives and two wine grapes varietals; river water samples (Plankenbrug River, Eerste River and Krom River) | Urinary tract infections, and wound infections; respiratory and urinary tracts of hospitalized adults and in the gastrointestinal systems of children | Lipopeptide | Cell-free serrawettin W1 homologues (serratamolide A with 515.33 m/z) and prodigiosin, glucosamine derivative A, and serrawettin W2 analogues (732.45 m/z) | Production after 120 h. The supernatants were lyophilized, resuspended in 70% (v/v) acetonitrile, and lyophilized for three times. Characterized by UPLC-MS analysis | Antimicrobial against *E. coli* ATCC 13706 (BS from P1 isolate), *L. longheachae* ATCC 33462 (BS from NP2 isolate); *L. pneumophilia* ATCC 33152 (BS from P1 and NP1); *P. aeruginosa* ATCC 27853 and S1 68 (BS from P1 and NP1), MDR strain *P. aeruginosa* PA3 (all the BS), *A. baumannii* ATCC 19606 (all the BS); XDR strain *A. baumanii* AB3 (BS from P1 and NP2); *B. cereus* ATCC 10876 (BS from P1 and NP1); *B. cereus* S1 77 (all the BS); *Bacillus* sp. S8 38 (BS from NP2); *E. faecalis* ATCC 7080 and S1 (BS from P1 and NP1); *L. monocytogenes* ATCC 13932 (BS from P1 and NP1); *L. monocytogenes* G1 (BS from P1 and NP2); *S. aureus* ATCC 25923 (BS from P1 and NP1); *S. equorum* SP2 (BS from P1); and MRSA strain *S. aureus* Xen 30 (BS from P1 and NP1); antifungal against *C. albicans* ATCC 66027, CAB8911, and CAB 1085 (BS from P1 and NP1); *C. neoformans* CAB 1055 and CAB 844 (BS from P1 and NP1); and *C. neoformans* CAB 1055 (all the BS); hemolytic activity | Possible broad-spectrum therapeutic agents against multidrug-resistant bacterial and fungal pathogens | Clements et al., 2019a |
